# Abnormal amphiregulin expression correlates with gastric cancer prognosis

**DOI:** 10.18632/oncotarget.12436

**Published:** 2016-10-04

**Authors:** Bing Wang, Hongmei Yong, Huijun Zhu, Daguang Ni, Sijie Tang, Shu Zhang, Wei Wang, Yan Zhou, Wei Zhao, Guipeng Ding, Jin Zhu, Xiaohua Li, Zhenqing Feng

**Affiliations:** ^1^ Center for Pathology and Laboratory Medicine, Zhangjiagang Ao Yang Hospital, Zhangjiagang, Jiangsu, China; ^2^ Department of Oncology, Zhangjiagang Ao Yang Hospital, Zhangjiagang, Jiangsu, China; ^3^ Department of Oncology, Huai'an Hospital Affiliated of Xuzhou Medical College and Huai'an Second People's Hospital, Huai'an, Jiangsu, China; ^4^ Department of Pathology, Affiliated Hospital of Nantong University, Nantong, Jiangsu, China; ^5^ Department of Pathology, Nanjing Medical University, Nanjing, China; ^6^ Huadong Medical Institute of Biotechniques, Nanjing, China; ^7^ School of Medicine, Jiangsu University, Jiangsu, China; ^8^ The Second Affiliated Hospital of Nanjing Medical University, Nanjing, China; ^9^ Key Laboratory of Antibody Technique of Ministry of Health, Nanjing Medical University, Nanjing, China; ^10^ Jiangsu Collaborative Innovation Center For Cancer Personalized Medicine, Nanjing Medical University, Nanjing, China

**Keywords:** gastric cancer, amphiregulin, prognosis

## Abstract

Gastric cancer (GC) is a global health issue with a high mortality rate. Early diagnosis and tracking of GC is a challenge due to a lack of reliable tools. Amphiregulin (AREG) is a member of the epidermal growth factor (EGF) family that activates growth signaling upon binding of EGF receptors. Elevated AREG expression is associated with various pathological conditions, including cancer. Here, we investigated whether increased AREG expression is a disease indicator and/or prognostic biomarker for GC. We used tissue microarray and quantitative real-time polymerase chain reaction to assess AREG expression in clinical tissue specimens at various stages of GC and a conducted bioinformatics analysis to evaluate the value of AREG over-expression as a GC biomarker. We found that both mRNA and protein expression of AREG were increased in the tissues of GC patients when compared to tissues from non-cancer patients or normal tissues. High expression of AREG was also associated with GC clinicopathological characteristics and poor survival. Thus, over-expression of AREG could serve as a novel GC biomarker, and active surveillance of its expression could be a novel approach to GC diagnosis and monitoring.

## INTRODUCTION

Despite its declining incidence in recent years, gastric cancer (GC) is still ranked as the fourth most common type of cancer and is the second leading cause of cancer-related death with a 20% 5-year survival rate. New incidences of stomach cancer in 2015 were estimated to number 24,590 (1.48% of total cancer cases), resulting in the deaths of 10,720 individuals (1.82% of a total of 1,658,370 cases; data from the AACR Cancer Progress Report, 2015). Occurrences of GC, particularly stomach cancer, are associated with many risk factors, including dietary factors and familial predisposition; however, chronic gastritis induced by Helicobacter pylori infection causes the majority of GC [1, 2]. Developing countries have higher incidences of stomach cancer, providing evidence that improved nutrition and sanitation could reduce GC rates [3]. In addition to these environmental risk factors, aberrant gene expression and germline mutations are also linked with higher GC incidence [4].

Endoscopy is an invasive operation with possible unpredictable side effects routinely used in the diagnosis of GC. Detection of tumor-associated molecules (CEA, CA19-9, CTSF, etc.) also aids in the diagnosis and/or prognosis of GC, but these biomarkers have shown limited clinical value due to low sensitivity and specificity [5]. Many novel molecules, including various proteins [6], autoantibodies against TAAs, cell-free DNA fragments, mRNAs, various non-coding RNAs, circulating tumor cells, and cancer-derived extracellular vesicles [5, 7], have been screened for their viability as cancer biomarkers. However, these efforts did not significantly improve clinical practice. Therefore, it is still urgent to identify new biomarkers for early GC diagnosis and/or for monitoring disease prognosis.

AREG is the shedding ectodomain of a 252 amino acid transmembrane precursor (pre-AREG) that is secreted into the blood or cellular microenvironment [8]. AREG is expressed by activated immune cells and is also constitutively expressed in many epithelial and mesenchymal cells [8]. As a growth factor, AREG activates growth-signaling pathways by binding to EGF receptors [9]. AREG plays a critical role in mammary gland development and branching morphogenesis of organs [10] and is a pro-oncogenic factor, as over-expression of AREG promotes malignant tissue development and disease progression. Over-expression of AREG in cancer cases is also associated with resistance to conventional chemotherapeutic agents (e.g. doxorubicin, cisplatin and sorafenib) [11–13]. However, there are few reports regarding the abnormal expression of AREG in GC patients [14, 15]. Here we report the results of tumor tissue microarray (TMA) analysis in combination with clinical investigation, and evaluate whether testing of AREG expression in GC tissue could be implemented as a predictive, diagnostic, and/or prognostic biomarker in GC.

## RESULTS

### *AREG* mRNA expression in gastric tissues

**Figure 1:**
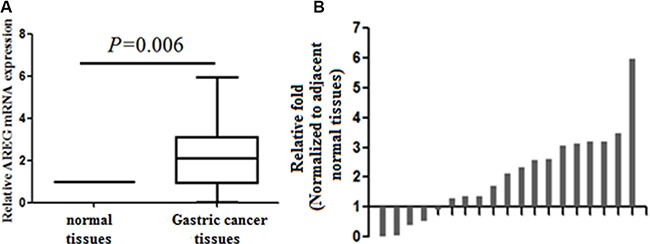
Analysis of *AREG* expression in gastric cancer tissues by qRT-PCR (**A**) Increased levels of *AREG* mRNA in 19 GC tissues. *ACTB* expression was used as an internal control and data are presented as relative fold change over *ACTB*. (**B**) *AREG* expression in individual GC tissues. Data are presented as the relative change in *AREG* expression in GC versus adjacent normal tissue in individual paired specimen.

Cycle threshold numbers were distributed from 32.6 to 36.8 for *AREG*, and from 18.4 to 19.5 for the *ACTB* in matched adjacent normal tissues, and the data are presented as relative fold change over *ACTB*. Average *AREG* expression in GC tissue was 2.04 ± 1.47 fold higher than in matched adjacent normal tissue (*p* = 0.006, Figure 1).

### *AREG* protein expression in benign and malignant gastric tissues

**Figure 2:**
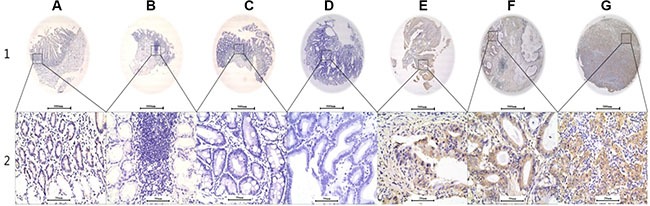
Representative AREG expression patterns in TMA sections, along with gastric cancer development from benign to malignant status Column (**A**) normal surgical margin of GC with low AREG expression (IHC score, 10); Column (**B**) chronic gastritis with no AREG expression (IHC score, 0); Column (**C**) intestinal metaplasia with no AREG expression (IHC score, 0); Column (**D**) low-grade intraepithelial neoplasia with no AREG expression (IHC score, 0); Column (**E**) high-grade intraepithelial neoplasia with high AREG expression (IHC score, 110); Column (**F**) well-differentiated GC with high AREG expression (IHC score, 160); Column (**G**) middle differentiated GC with high AREG expression (IHC score, 210). AREG staining was reviewed at 40× magnification in Row 1, and at 400× magnification in Row 2.

Total 817 (94.89%) of the 861 specimens were analyzed for AREG expression on the TMA. The other 44 tissues were lost during antigen retrieval, which is acceptable for the analysis of TMA [16, 17]. Clinical data from the 817 specimens are summarized in Table 1. Clinicopathological data collected from 592 patients with primary GC are summarized in Table 2. The average age was 58.5 years old (range, 31~85 years old). IHC staining (Figure 2) revealed that AREG was expressed in both the cell membrane and cytoplasm, consistent with previous reports [14, 15]. AREG staining in most of normal tissues (76.92%) was categorized as “no or low”, and expression was increased in tumor tissue, which was consistent with the *AREG* mRNA expression data (Figure 1). AREG protein expression of “High” was recorded as 39.30% of the stomach benign tissues in chronic gastritis, 37.93% of the intestinal metaplasia tissues, 30.0% of the low-grade intraepithelial neoplasia tissues, 37.50% of the high-grade intraepithelial neoplasia tissues, and 23.08% of the matched non-tumor tissues. In contrast, 66.72% of GC tissue samples had “High” AREG expression, which was significantly higher than in benign tissues (Table 1; *p <* 0.001). Thus, over-expression of AREG in stomach tissue may serve as a GC diagnostic biomarker.

**Table 1:** AREG expression in gastric tissues

Characteristic	*n*	Patients number of AREG expression (%)		Pearson χ^2^	*p*
		No or Low	High		
				**89.723**	< 0.001*
**Stomach**					
Chronic gastritis	66	40 (60.61)	26 (39.39)		
Intestinal metaplasia	29	18 (62.07)	11 (37.93)		
Low-grade intraepithelial neoplasia	10	7 (70,00)	3 (30.00)		
High-grade intraepithelial neoplasia	16	10 (62.50)	6 (37.50)		
Cancer	592	197 (33.28)	395 (66.72)		
Matched tumor neighbor	104	80 (76.92)	24 (23.08)		

* *P* < 0.05.

**Table 2:** Association of high expression of AREG with clinicopathologic characteristics in gastric cancer patients

Characteristic	*n*	AREG expression (%)		Pearson χ^2^	*p*
		No or Low	High		
Total	591	198 (33.50)	393 (66.50)		
**Gender**				0.324	0.320
Male	421	144 (34.20)	277 (65.80)		
Female	170	54 (31.80)	116 (68.20)		
**Age**				1.914	0.098
< 60	328	102 (31.10)	226 (68.90)		
≥ 60	263	96 (36.50)	167 (63.50)		
**Histological type**				14.289	0.006*
Tubular	517	173 (33.50)	334 (66.50)		
Mixed (tubular and mucinous)	7	1 (14.3.60)	6 (85.70)		
Mucinous	33	9 (27.30)	24 (72.70)		
Signet ring cell	22	13 (59.10)	9 (40.50)		
Others^a^	12	0 (0.00)	12 (100.00)		
**Differentiation**				8.185	0.042*
Well	57	27 (47.40)	30 (52.60)		
Middle	141	51 (36.20)	90 (63.80)		
Poor	326	95 (29.10)	231 (70.90)		
Others^b^	67	23 (34.30)	44 (65.70)		
**TNM stage**				24.061	< 0.001*
0 + I + II	329	137 (41.60)	192 (58.40)		
III + IV	262	59 (22.50)	203 (77.50)		
**Tumor stage**				35.034	< 0.001*
T0	18	10 (50.60)	8 (44.40)		
T1 + T2	176	86 (48.90)	90 (51.10)		
T3 + T4	397	100 (25.20)	297 (74.80)		
**Lymph node metastases**				15.365	< 0.001*
N0	221	95 (43.00)	126 (57.00)		
N1 + N2 + N3	370	101 (27.30)	269 (72.70)		
**Distant metastases**				9.389	0.001*
M0	553	192 (34.70)	361 (65.30)		
M1	38	4 (10.50)	34 (89.50)		

a others: papillary adenocarcinoma,4 cases; Adeno-squamous carcinoma,4 cases; Squamous cell carcinoma, 2cases; Undifferentiated carcinoma, 2 cases; Neuroendocrine carcinoma, 1 cases.

b others: besides tubular and papillary adenocarcinoma.

* *P* < 0.05.

### Association of *AREG* expression with clinicopathological characteristics in gastric cancer

The correlations between AREG expression and clinicopathological variables in patients are summarized in Table 2. There was a correlation between increasing levels of AREG expression and the progression of tissue malignancy. Additionally, the proportion of patients with “high” AREG expression was associated with disease progression, including TNM stage (*p <* 0.001), invasion (*p <* 0.001), lymph node metastasis (*p <* 0.001), distant metastasis (*p* = 0.001), histological type (*p* = 0.006), and there was a trend of a correlation with de-differentiation (*p* = 0.042). These results demonstrated that the change in AREG expression reflected the patient's symptoms and disease progress. No correlations were found between AREG expression and age or gender (Table 2).

### Increased *AREG* expression correlates with poor overall survival

**Figure 3:**
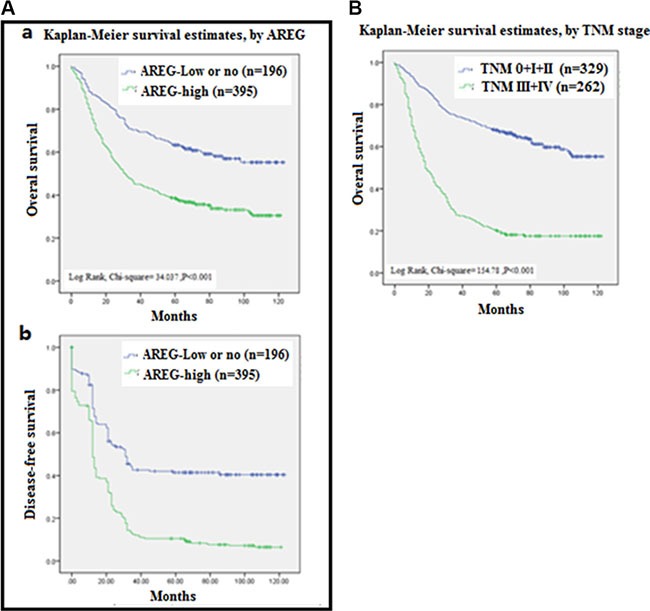
Analysis of Survival curves for patients with gastric cancer (**A**) Overall survival (OS) curves (a) and Disease free survival curves (b) of patients with “high” AREG expression (green line, 1) and with “no or low” AREG expression (blue line, 2) were analyzed by Kaplan-Meler survival and log-rank test. (**B**) OS curves of patients with different Tumor Node Metastasis (TNM) stages of 0 + I + II (blue line, 1), III + IV (green blue line, 2) were analyzed by Kaplan-Meler survival and log-rank test.

In univariate analysis, AREG expression (HR 2.734, 95% CI 1.923–3.889; *p <* 0.001) was significantly associated with poor OS, as well as de-differentiation (HR 1.321, 95% CI 1.075–1.624 *p* = 0.008) and TNM stage (HR 7.370, 95% CI 5.010–10.842; *p <* 0.001). In multivariate analysis, only “high” AREG expression and TNM stage remained significantly associated with poor OS (HR 2.143, 95% CI 1.454–3.159; *p <* 0.001 and HR 6.570, 95% CI 4.427–9.750; *p <* 0.001, respectively; Table 3; Figure 3). These data further demonstrate that increased AREG expression could be a prognostic biomarker for GC.

**Table 3:** Univariate and multivariate analysis of prognostic markers for overall survival in gastric cancer

	Univariate analysis			Multivariate analysis		
	HR	*p* > | z |	95%CI	HR	*p* > | z |	95%CI
**AREG expression**						
High *vs* Low or no	2.734	< 0.001*	1.923–3.889	2.143	< 0.001*	1.454–3.159
**Age**						
< 60 *vs* ≥ 60	1.067	0.725	0.743–1.531	—	—	—
**Gender**						
Male *vs* Female	0.975	0.911	0.630–1.510	—	—	—
**Histological type**						
Tubular *vs* Mixed(tubular and mucinous)*vs* Mucinous *vs* signet ring cells *vs* others^a^	0.934	0.462	0.779–1.120	—	—	—
**Differentiation**						
Well *vs* Middle *vs* Poor	1.321	0.008*	1.075–1.624	1.083	0.501	0.859–1.366
**TNM stage**						
0 + I + II *vs* III + IV	7.370	< 0.001*	5.010–10.842	6.570	< 0.001*	4.427–9.750
**Tumor stage**						
T0 *vs* T1 + T2*vs* T3 + T4	4.530	< 0.001*	3.194–6.426	—	—	—
**Lymph node metastases**						
N0 *vs* N1 + N2 + N3	4.658	< 0.001*	3.260–6.655	—	—	—
**Distant metastases**						
M0 *vs* M1	6.811	< 0.001*	2.385–9.454	—	—	—

a others: papillary adenocarcinoma,4 cases; Adeno–squamous carcinoma,4 cases; Squamous cell carcinoma, 2cases; Undifferentiated carcinoma,2 cases; Neuroendocrine carcinoma, 1 cases.

* *P* < 0.05.

## DISCUSSION

A histopathological model of GC development suggests that GC develops sequentially from normal mucosa to chronic gastritis then to chronic atrophic gastritis, intestinal metaplasia, and dysplasia (including low-grade to high-grade intraepithelial neoplasia), and finally to adenocarcinoma [18, 19]. The turning point of the malignant tissue/cell changes is in the status of intestinal metaplasia [20]. While many factors are involved in the progression of GC, aberrant expression of epidermal growth factor receptor (EGFR) and its cognate ligands (e.g. EGF and AREG) is one of the major causes for malignancy progression and cancer formation [21]. As a pro-oncogenic molecule, aberrant expression of AREG could promote abnormal activation of cell signaling transduction and subsequent gene transcription, which, in turn, could lead to cancer development and progression [22–24]. As an ectodomain of transmembrane glycoprotein of precursor, AREG is over-expressed in the epithelial tissue of gastric [25], pancreatic [26], colon, and prostate cancer [27], as well as renal cell carcinoma [28]. After proteolytic cleavage, the shedding ectodomain of pro-AREG is secreted into the blood and other body fluids as a soluble growth factor [8]. Thus, AREG expression in body fluid could reflect cellular AREG expression and, in turn, serve as biomarker for tissue malignancy [29].

High levels of serum AREG have been shown to activate AKT and ERK signaling and promote early disease progression in cancer patients [30]. High concentrations of AREG in malignant ascites of GC patients also have been found to play an important role in the development of peritoneal carcinomatosis via interactions with CXCL12/CXCR4, suggesting that the AREG/CXCL12/CXCR4 axis could be a potential therapeutic target for peritoneal carcinomatosis of GC [31]. In a cellular model, AREG acted as an autocrine growth factor and evoked the induction of growth signaling to promote tumor cell proliferation and tumor angiogenesis [15]. Thus, AREG might serve as a master regulator and participate in the alteration of multiple cellular events related with tumor progression. Indeed, in epithelial cancer development, AREG was shown to activate forkhead box protein M1 (FoxM1) to alter the expression of 623 genes, in turn deregulating G2/M progression and cytokinesis and promoting malignant cell proliferation [32]. Taken together, the literature suggests that AREG-activated aberrant signaling pathways could be a target for cancer therapy.

AREG expression has also been found to reduce chemosensitivity and impact the efficacy of drug interventions [33–35]. This AREG-mediated drug resistance has also been associated with aberrant cell signaling. For instance, AREG was shown to activate AKT and ERK pathways, resulting in tumor cell proliferation and the reduced efficacy of trastuzumab treatment [30]. Similarly, abnormal activation of WNT signaling enhanced AREG expression, leading to Gefitinib resistance [11]. In addition, using the tet-off system to manually control the expression of 11 amino acids of AREG protein at the C-terminus and thereby promote the translocation of pro-AREG from the plasma membrane to the nucleus, it was found that nuclear pro-AREG increased the resistance of cells to anti-cancer drugs [13]. Consequently, down regulation of AREG with siRNA increased the number of apoptotic cells [28]. However, it has also been reported that *AREG* gene expression was associated with good outcomes after the curative resection of stage II/III GC [36]. These conflicting results indicate that the biological functions of AREG are complex. Thus, the role of AREG over-expression in GC should be carefully addressed in the context of individual diseases.

Malignant transformation in digestive tissues is commonly seen in the Asian population. Thus, it is critical to study and develop effective tools for its early diagnosis and to improve treatment [37, 38]. Previous studies found that over-expression of AREG could serve as a cancer biomarker in a various cancer types, but its role in GC progress was still undefined [39]. In this study, we showed that AREG expression at both the mRNA and protein levels was higher in GC patients when compared with non-cancer patients and normal tissues. Analysis by IHC also revealed that increasing levels of AREG protein correlated with the stage of cancer development and progress. Clinical investigation also indicated that increased AREG expression was associated with tumor progression including TNM, invasion, and metastasis, and was correlated with poor survival. Our findings suggest that AREG could serve as a GC biomarker and that surveillance of AREG expression could be an effective approach for GC diagnosis and tracking. High expression of AREG may also be considered a promising target for cancer chemotherapy.

## MATERIALS AND METHODS

### Human tissue specimens and patient clinical information

A total of 817 formalin-fixed paraffin-embedded stomach tissue specimens were collected from the department of Pathology, Affiliated Hospital of Nantong University from 2003 to 2010. These included 66 chronic gastritis tissues, 29 intestinal metaplasia tissues, 10 low-grade intraepithelial neoplasia tissues, 16 high-grade intraepithelial neoplasia tissues, 592 cancer tissues, and 127 matched tumor-adjacent normal tissues. An additional set of 24 freshly frozen GC tissues and matched tumor-adjacent normal tissues were obtained from the first Affiliated Hospital of Nanjing Medical University, Huai'an Second People's Hospital and Zhangjiagang AoYang Hospital. The study protocol was approved by the Human Research Ethics Committee of the hospital. Clinical data were extracted from patients’ medical records, including age, sex, Tumor Node Metastasis (TNM) stage, histological type, and differentiation status. None of the cancer patients received any type of treatment (such as radiation therapy, chemotherapy, or immunotherapy) before surgery. Overall survival (OS) was defined as the period of time from initial biopsy diagnosis to death. Disease-free survival (DFS) was defined as the period from follow-up to recurrence. The follow-up process ranged from 2 to 10 years, and patients who were alive at the last date of follow-up were included for data analysis.

### Tissue microarray (TMA) construction and immunohistochemistry (IHC)

TMA was generated following the instructions of the Tissue Microarrayer System Quick Ray (UT06, UNITMA, Korea). Briefly, individual formalin-fixed paraffin-embedded blocks were placed in a new recipient paraffin block to contract the core tissue biopsies to 2 mm in diameter. A total of 13 gastric TMAs were generated by cutting four-micron sections of prepared core tissue biopsies and placing them on super frost-charged glass microscope slides. When conducting IHC staining, the TMA tissue sections were de-paraffinized and rehydrated with graded alcohols. Then endogenous peroxidase activity was blocked by incubating the slides in 3% H_2_O_2_. Antigen retrieval was carried out with 0.01 M citrate buffer (pH 6.0) and microwave heat induction. Goat anti-human AREG polyclonal antibody (dilution 1:100, R&D Systems, AF262) was applied to detect AREG expression. Reactions were examined with an Envision+™ peroxidase kit (Dako, Carpinteria, CA, USA) after incubation with 3, 3′-diaminobenzidine plus (Dako, Carpinteria, CA, USA). Slides were then counterstained with Hematoxylin and dehydrated with graded alcohols, cleared in xylene, covered with coverslips, and sealed with permanent mounting media.

All slides were reviewed by a pathologist blind to the patient's clinical characteristics. AREG expression was scored using the semi-quantitative H-score method by taking into account both the staining intensity and the percentage of cells at that intensity [40]. The result of staining intensity was scored as 0 (no staining), 1+ (weak staining), 2+ (moderate staining), or 3+ (intense staining). For each of the four staining intensity scores, the percentage of cells stained at the respective intensity was determined and multiplied by the intensity score to yield an intensity percentage score. The final staining scores were then calculated from the sum of the four intensity percentage scores. Thus, the resulting staining score had a minimum value of 0 (no staining) and a maximum of 300 (100% of cells with 3+ staining intensity). A cutoff score in tissue staining was determined as 100 in terms of OS of the patient prior to the final analysis (described later on). A score between 0 and 130 was considered as “no or low” protein expression, while a score between 131 and 300 was considered as “high” protein expression.

### Quantitative real-time polymerase chain reaction (qRT-PCR)

Tissue specimens (19 pairs of human GC and their adjacent tissues) were collected, snap-frozen in liquid nitrogen, and stored at −80°C before RNA extraction. Total RNA was extracted from frozen samples using Trizol reagent (Invitrogen, Carlsbad, CA, USA). The 260/280 ratios of isolated RNA samples were all between 1.8~2.0 and the RNA quality confirmed with the appearance and ratio of 28s and 18s fragments via agarose gel electrophoresis. Two μg of total RNA was used to generate cDNA by reverse transcription with a PrimeScript™ RT reagent kit (Takara, Glen Burnie, MD) following the manufacturer's instructions. qRT-PCR analysis was performed to determine *AREG* mRNA expression with *ACTB* expression serving as the internal control for normalization and quantification. The primers to amplify human *ACTB* were as follows: forward, 5′- TGGAGAAAATCTGGCACCAC-3′ and reverse, 5′-GATGATGCCTCGTTCTAC-3′, and the *AREG* primers were: forward, 5′-GCTGTCGCTCTTGATAC TCG-3′, and reverse,5′-ACGCTTCCCAGAGTAGGT GT-3′(Genescript. Nanjing, China).

The qRTPCR reaction was conducted at a final volume of 20 μL containing 2 μL of cDNA template (corresponding to ~40 ng of retro-transcribed total RNA), 20 nmol/L of each primer, and 2× SYBR Green PCR Master Mix (10 μL; Applied Biosystems). PCR amplification was performed in an ABI PRISM 7500HT Sequence Detection System (Applied Biosystems, Foster City, CA, USA) in 96-well plate format. After an initial 2 min hold at 50°C to allow AmpErase-UNG activity and 10 min at 95°C, amplification was performed at 95°C for 15 sec and 58°C for 1 min for 40 cycles. Samples were run in triplicate, Ct values were collected, and *AREG* mRNA levels for each sample were calculated using the 2^−ΔΔCt^ method [41–43].

### Statistical analysis

Data were analyzed using SPSS 18.0 statistical software (SPSS Inc., Chicago, IL). Student's *t* test and Pearson χ^2^ tests were performed to determine if there was a statistically significant difference between groups. The X-tile software program (The Rimm's Lab at Yale University; http://medicine.yale.edu/lab/rimm/research/software.aspx) was used for statistical analysis of the IHC data after it was converted into binary data (“no or low” versus “high”) using pre-determined cutoff values [44]. Both the Kaplan-Meier method and a log-rank test were implemented to evaluate if there was a significant difference in the OS of the patients. The univariate and multivariate hazard ratios for the variables were analyzed by a Cox proportional hazards model. A *p-value* of less than 0.05 was considered statistically significant.
